# Pyramid-Net: Intra-layer Pyramid-Scale Feature Aggregation Network for Retinal Vessel Segmentation

**DOI:** 10.3389/fmed.2021.761050

**Published:** 2021-12-07

**Authors:** Jiawei Zhang, Yanchun Zhang, Hailong Qiu, Wen Xie, Zeyang Yao, Haiyun Yuan, Qianjun Jia, Tianchen Wang, Yiyu Shi, Meiping Huang, Jian Zhuang, Xiaowei Xu

**Affiliations:** ^1^Guangdong Provincial Key Laboratory of South China Structural Heart Disease, Guangdong Provincial People's Hospital, Guangdong Cardiovascular Institute, Guangdong Academy of Medical Sciences, Guangzhou, China; ^2^Shanghai key Laboratory of Data Science, School of Computer Science, Fudan University, Shanghai, China; ^3^Department of Computer Science and Engineering, University of Notre Dame, Notre Dame, IN, United States; ^4^Oujiang Laboratory (Zhejiang Lab for Regenerative Medicine, Vision and Brain Health), Wenzhou, China; ^5^Cyberspace Institute of Advanced Technology, Guangzhou University, Guangzhou, China; ^6^College of Engineering and Science, Victoria University, Melbourne, VIC, Australia

**Keywords:** deep learning, neural network, feature aggregation, pyramid scale, retinal vessel segmentation

## Abstract

Retinal vessel segmentation plays an important role in the diagnosis of eye-related diseases and biomarkers discovery. Existing works perform multi-scale feature aggregation in an inter-layer manner, namely **inter-layer feature aggregation**. However, such an approach only fuses features at either a lower scale or a higher scale, which may result in a limited segmentation performance, especially on thin vessels. This discovery motivates us to fuse multi-scale features in each layer, **intra-layer feature aggregation**, to mitigate the problem. Therefore, in this paper, we propose Pyramid-Net for accurate retinal vessel segmentation, which features intra-layer pyramid-scale aggregation blocks (IPABs). At each layer, IPABs generate two associated branches at a higher scale and a lower scale, respectively, and the two with the main branch at the current scale operate in a **pyramid-scale** manner. Three further enhancements including pyramid inputs enhancement, deep pyramid supervision, and pyramid skip connections are proposed to boost the performance. We have evaluated Pyramid-Net on three public retinal fundus photography datasets (DRIVE, STARE, and CHASE-DB1). The experimental results show that Pyramid-Net can effectively improve the segmentation performance especially on thin vessels, and outperforms the current state-of-the-art methods on all the adopted three datasets. In addition, our method is more efficient than existing methods with a large reduction in computational cost. We have released the source code at https://github.com/JerRuy/Pyramid-Net.

## 1. Introduction

The subtle changes in the retinal vascular, including vessel width, tortuosity, and branching features, indicate mass eye-related diseases, such as diabetic retinopathy (1), glaucoma (2), and macular degeneration (3). Meanwhile, those characteristics are important biomarkers for numerous systemic diseases, including hypertension (4) and cardiovascular diseases (5). Retinal vessel segmentation is one of the cornerstones to access those characteristics, particularly for automatic retinal image analysis (6, 7). For example, hypertensive retinopathy is a retinal disease, which is caused by hypertension. Increased vascular curvature or stenosis can be found in patients with hypertension (8). Conventionally, manual segmentation is laborious and time-consuming, and suffers subjectivity among experts. To improve efficiency and reliability and reduce the workload of doctors, the clinical practice puts forward high requirements for automatic segmentation (9).

**Figure 1:**
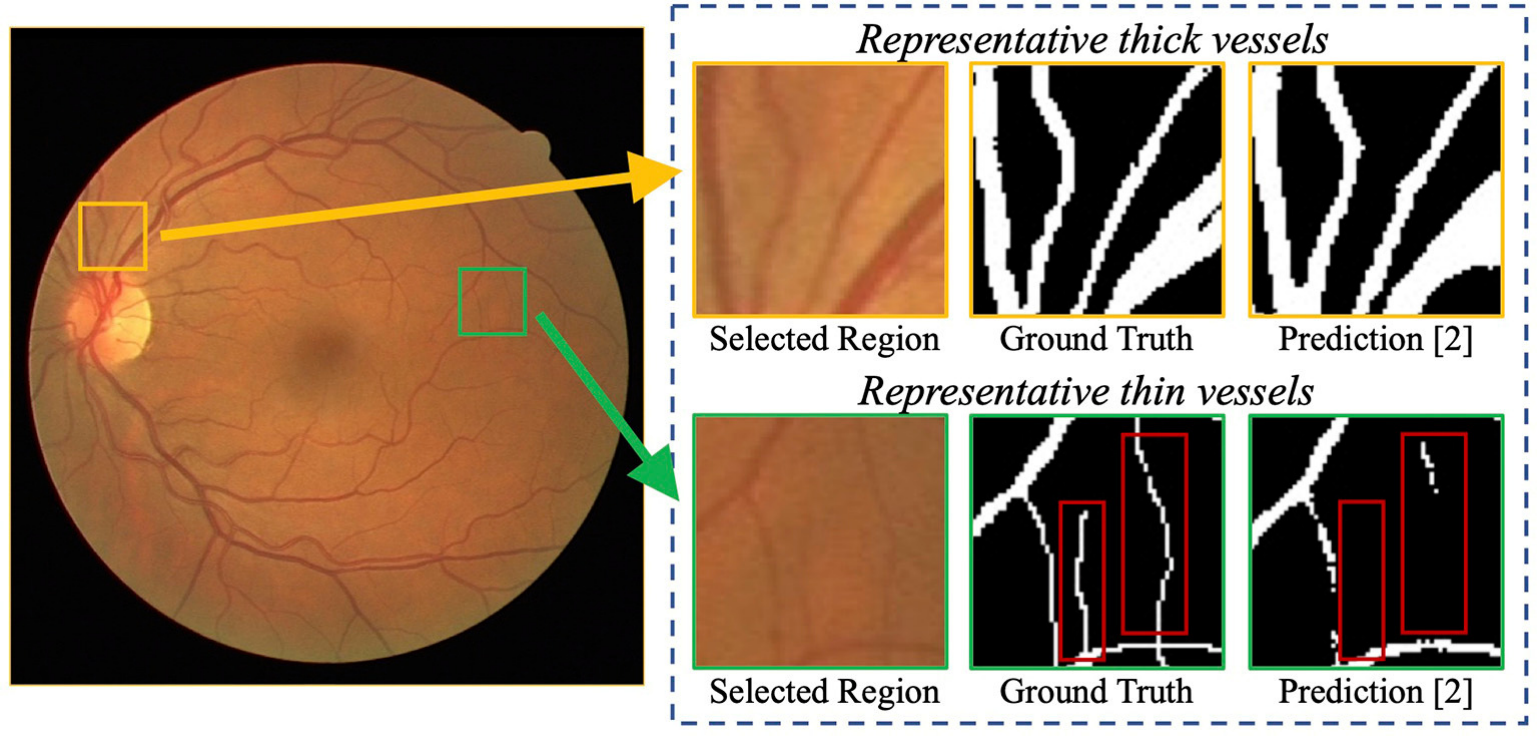
Examples of challenging thin vessels in retinal vessel segmentation. The retinal fundus image (left) contains numerous thin vessels (1–2 pixels wide) and thick vessels (3 pixels wide or more) (10). Regions of representative thin and thick vessels, and their corresponding ground truth and predictions (11) are shown in the right. It can be noticed that the thick vessels obtain a better segmentation performance, while the thin vessels suffer a big miss (indicated by red rectangles).

Recently, deep neural networks have boosted the segmentation performance of retinal vessel segmentation (10, 12) by a large margin compared with traditional methods (13, 14). However, thin vessels cannot be segmented accurately. For example, Figure 1 demonstrates a commonly-seen fundus image containing numerous thin vessels and thick vessels, and corresponding segmentation (11) and ground truth. We can easily notice that the thick vessels enjoy a promising performance, but the thin vessels suffer a big miss. A potential reason is that the continuous pooling operations in most neural networks are used to encode the features, which leads to a mass loss of appearance information and harms the segmentation accuracy, especially on thin vessels. Note that in practice, it is also difficult to segment these thin vessels for experts due to low contrast and ambiguousness. Currently, some works have been proposed to tackle the above problems, e.g., a particular processing branch for thin vessels (12), a new loss function to emphasize thin vessels (10). However, the segmentation performance is still limited considering the clinical requirement of retinal image analysis.

Meanwhile, **multi-scale feature aggregation** to fuse coarse-to-fine context information has been popular to segment thin/small objects (15–19). There are mainly two approaches: input-output level category and intra-network level category. In the input-output level category, connections exist between inputs at various scales and corresponding intermediate layers (15), or between the intermediate layers and the final predictions with corresponding scales (18). In the intra-network level category, features from previous layers are adjusted in channel numbers and spatial dimension and then aggregated with the ones in the later layer (16). However, current multi-scale feature aggregation works in an inter-layer manner, **inter-layer feature aggregation**, which can only fuse features at either a lower scale or a higher scale. For example, in the encoder, feature maps at the lower scale cannot be fused by that at the current scale because of the processing order of the layers. A possible solution is to fuse multi-scale features in each layer, **intra-layer feature aggregation**, to consider features at both the high scale and the low scale.

Motivated by the above discoveries, in this paper, we propose Pyramid-Net for accurate retinal vessel segmentation. In each layer of Pyramid-Net, intra-layer pyramid-scale aggregation blocks (IPABs) are employed in both the encoder and the decoder to aggregate features at pyramid scales (the higher scale, the lower scale, and the current scale). In this way, two associated branches at the higher scale and the lower scale are generated to assist the main branch at the current scale. Therefore, coarse-to-fine context information is shared and aggregated in each layer, thus improving the segmentation accuracy of capillaries. To further improve the performance, three optimizations, including pyramid inputs enhancement, deep pyramid supervision, and pyramid skip connections, are applied to IPABs. We have conducted comprehensive experiments on three retinal vessel image segmentation datasets, including DRIVE (20), STARE (21), and CHASE-DB1 (22) with various segmentation networks. The experimental results show that our method can significantly improve the segmentation performance, especially on thin vessels, and achieves state-of-the-art performance on the three public datasets. In addition, our method is more efficient than the existing method with a large reduction in computational cost.

Overall, this work makes the following contributions:

1) We discovered that thin vessels suffer a big miss in the segmentation results of existing methods;
2) We proposed Pyramid-Net for retinal vessel segmentation in which intra-layer pyramid-scale aggregation blocks (IPABs) aggregate features at the higher, current, and lower scales to fuse coarse-to-fine context information in each layer;
3) We further propose three enhancements: pyramid input enhancement, deep pyramid supervision, and pyramid skip connections to boost the performance;
4) We conducted comprehensive experiments on three public vessel image datasets (DRIVE, STARE, and CHASE-DB1), and our method achieves the state-of-the-art performance on three datasets.

The remainder of this paper is organized as follows. Section 2 introduces related works and the motivation of the proposed method. Section 3 details the overall framework of the proposed Pyramid-Net, including IPABs and three optimizations (pyramid inputs enhancement, deep pyramid supervision, and pyramid skip connections). Section 4 first introduces datasets, implementation, and evaluation. Second, quantitative evaluations on three vessel image datasets, comparisons with the state-of-the-art algorithms, and several visual retinal segmentation results are presented. Third, several ablation studies that included evaluating the thin vessel, ablation analysis, and cross-training evaluation are discussed. Section 5 concludes the paper.

## 2. Related Work and Motivation

### 2.1. Vessel Image Segmentation

With the emergence of numerous public-available retinal image datasets (20–22), the supervised vessel segmentation methods became popular in the community. Commonly-seen supervised methods consist of two steps: feature extraction and classification. Some methods extracted the color intensity (24) and principle components (25) from the images, while some methods utilized wavelet (26) and edge responses (27). In terms of classification, various classic classifiers, including Support Vector Machine (SVM) (28), perceptron (29), random decision forests (30), and Gaussian model (26) are commonly seen and widely used in traditional supervised vessel image segmentation. Recently, in the light of fully convolutional networks (FCNs) (31) and U-Net (23), data-driven deep learning-based methods have demonstrated promising results and dominated the area of vessel image segmentation. Yan et al. (10) pointed out that the training loss tends to ignore the loss of thin vessels and is dominated by the thick vessels, which may be caused by the imbalance between thin vessels and thick vessels. Furthermore, Yan et al. (12) explored a three-stage network separating the segmentation of thick vessels, thin vessels, and the vessel fusion into different stages to make full use of the difference between thick and thin vessels to improve the overall segmentation performance. Considering that the consecutive pooling may lead to accuracy loss, CE-Net (32) encodes the high-dimension information and preserves spatial information to improve the overall segmentation. HA-Net (33) dynamically assigns the regions in the image hard regions or simple regions, and then introduces attention modules to help the network concentrate on the hard region for accurate vessel image segmentation. Meanwhile, some works introduce the spatial attention (34) and the channel attention (34) to the vessel segmentation domain and achieve promising results. The proposed method extends considerably to our previous work (35), which only supply some simplified evaluation on two public available vessel segmentation datasets. In this work, we have added a new module named “pyramid skip connections,” which furthers boost the performance. Meanwhile, we have added another widely-used dataset (STARE) to demonstrate the generalization of our proposed Pyramid-Net. Moreover, in terms of the analysis, we have supplied in-depth analyses of our method including evaluation on thin vessel segmentation, ablation analysis, and cross-training evaluation.

### 2.2. Motivation

**Figure 2:**
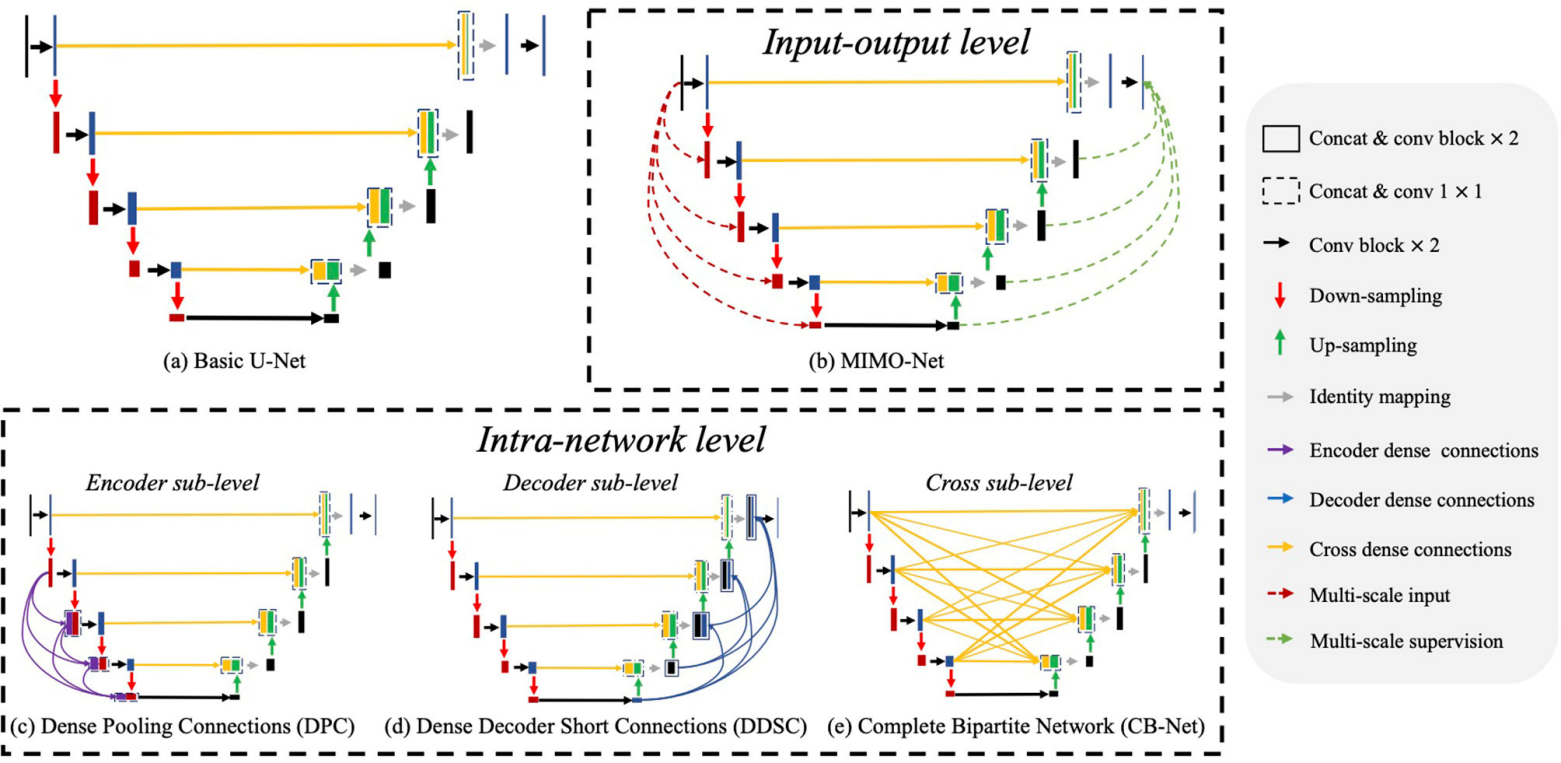
Illustrations of network structures of **(a)** basic U-Net (23) and **(b–e)** existing multi-scale feature aggregation methods, which mainly consist of two major categories: input-output level and intra-network level. The input-output level category means that the network employs multiple scaled inputs, and the scaled ground truth supervises the inter feature maps. In the intra-network level category, the encoder level, the decoder level, and the cross-level indicate implemented multi-scale feature aggregation in the encoder, the decoder, and their cross, respectively.

Multi-scale feature aggregation is widely used in medical image segmentation, which fuses the previous feature maps with different scales to improve the network performance. As shown in Figure 2, recent works (36–39) introduced multi-scale feature aggregation to strengthen feature propagation, alleviate the vanishing gradient problem, and improve the overall segmentation. We divide those methods into two major categories: input-output level and intra-network level.

**Input-output level category**: The connections exist between inputs at various scales and corresponding intermediate layers, or between the intermediate layers and the final predictions with corresponding scales. For example, Wu et al. (40) generated multi-scale feature maps by max-pooling and up-sampling layer and employed two sub-models to extract and aggregate features at multiple scales. MIMO-Net (41) fused scaled input images with multiple resolutions into the intermediate layers of the network in the encoder, and optimized the features in the decoder to improve the overall segmentation performance. MILD-Net (42) fused scaled original images with multiple resolutions to alleviate the potential accuracy decline caused by max-pooling.

**Intra-network level category**: In this approach, features from previous layers are adjusted in channel numbers and spatial dimension and then aggregated with the ones in the later layer. For ease of discussion, we discuss the network structures of related works based on the U-Net as shown in Figure 2. Note that U-Net is the most widely-used network in medical image segmentation. These works contain three main approaches: dense connections in the encoder (encoder sub-level), dense connections in the decoder (decoder sub-level) and dense connections in the cross of the encoder and the decoder (cross sub-level): (1) Encoder sub-level: (15) aggregated the scale inputs into the intermediate layers in the encoder to alleviate the accuracy decline caused by pooling; (2) Decoder sub-level: Dense decoder short connections (18) made full use of the feature maps in the decoder by fusing them with the feature maps in later layers; (3) Cross sub-level: Complete bipartite networks (16) inspired by the structure of complete bipartite graphs connected every layer in the encoder and the decoder.

Though multi-scale feature aggregation can significantly improve segmentation performance, we discover that they usually work in an inter-layer manner, **inter-layer feature aggregation**. In such a manner, features at either a lower scale or a higher scale are fused by the current layer. For example, in the encoder, feature maps at the lower scale cannot be fused by that at the current scale because of the processing order of the layers. The same phenomenon also exists in the decoder. Note that a successful segmentation needs to consider both feature maps at high scales for global localization information and low scales for detailed appearance information. Thus, we may mitigate the above problem by performing multi-scale feature aggregation in each layer of the network, **intra-layer feature aggregation**. How to obtain the multi-scale features in each layer becomes another problem. We may use pooling and upsampling to obtain two associated branches operating on a higher scale and a low scale, respectively. In this way, there exist three branches at three different scales (namely **pyramid scales**) in each layer, which is like a ResNet block (43). In this way, we may aggregate coarse-to-fine context information from pyramid-scale feature maps in each layer to further improve the segmentation performance.

## 3. Methods

**Figure 3:**
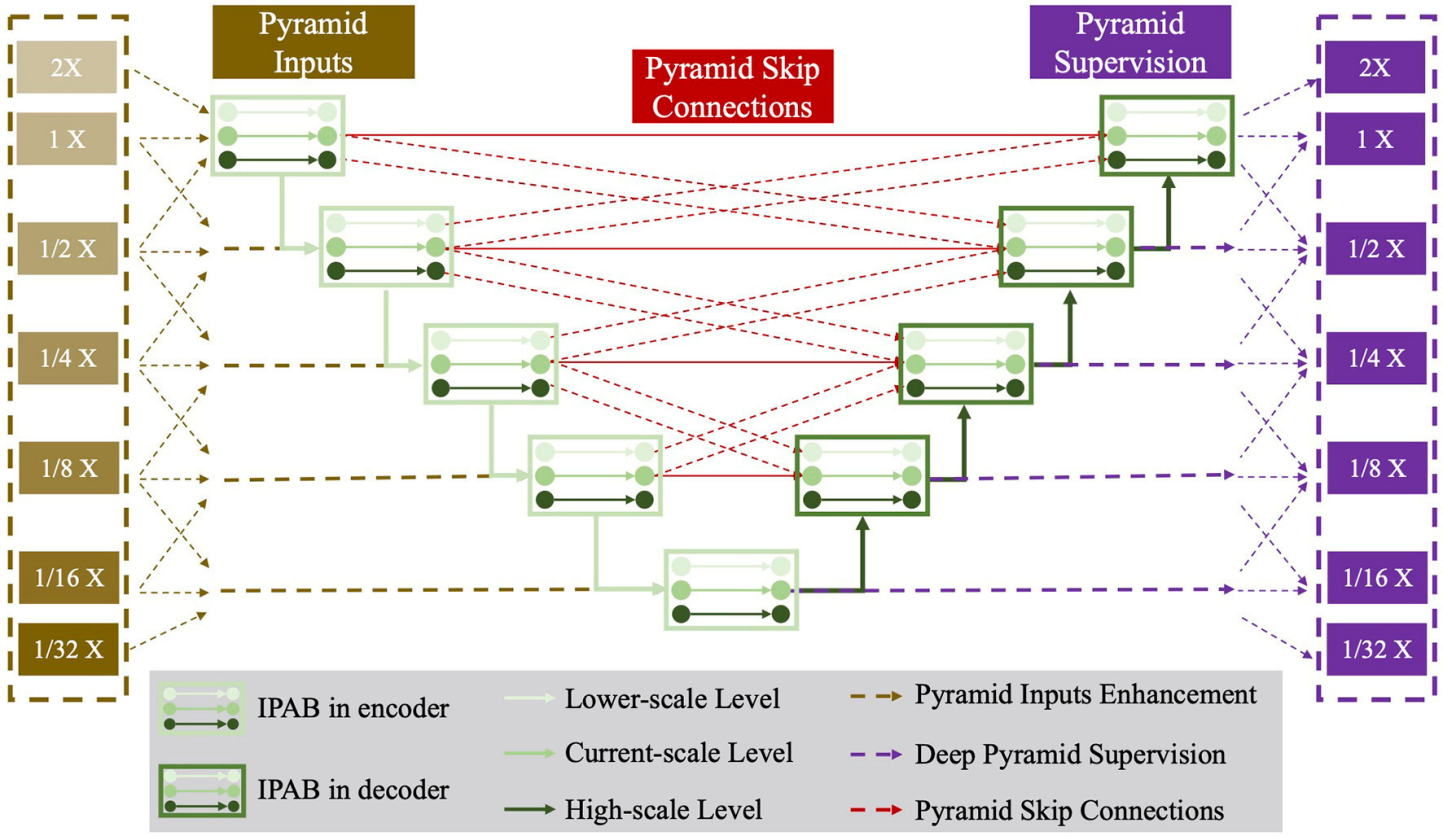
The network structure of the proposed Pyramid-Net. IPABs (green rectangle) not only aggregate features at **pyramid scales** [the current scale (green line), the higher scale (dark green line) and the lower scale (bright green line)] containing coarse-to-fine context information. Meanwhile, pyramid input enhancement (yellow rectangle), deep pyramid supervision (purple rectangle), and pyramid skip connections (rad rectangle) are employed to further improve the overall segmentation. Best viewed in color.

In this section, we first introduce IPABs and then describe three optimizations, including pyramid input enhancement, deep pyramid supervision, and pyramid skip connections. Figure 3 presents the structure details of Pyramid-Net.

### 3.1. Intra-layer Pyramid-Scale Aggregation Block

**Figure 4:**
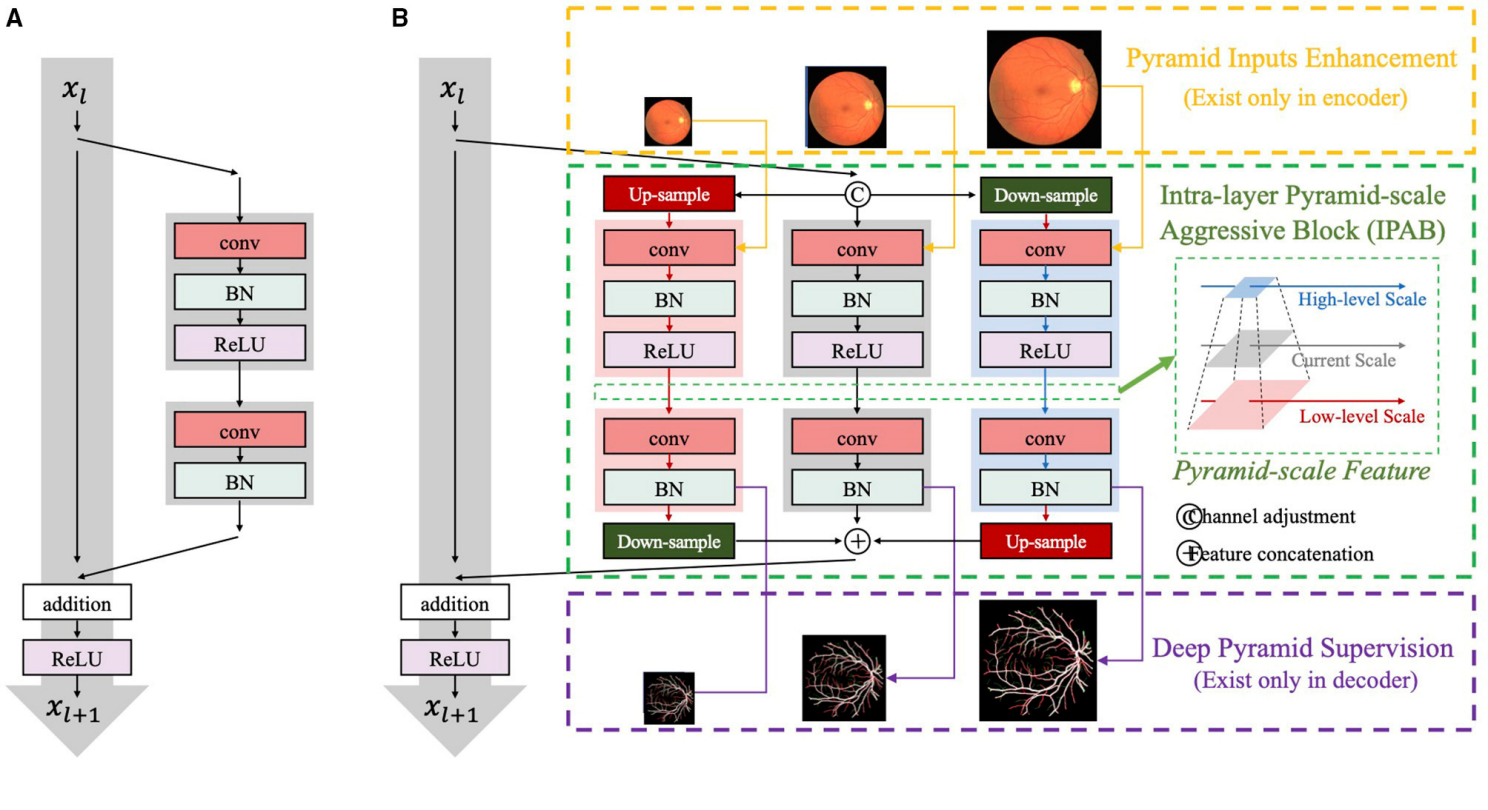
The network structure of **(A)** ResNet blocks and **(B)** our intra-layer pyramid-scale aggregation blocks (IPABs). IPABs (marked by green rectangles) aggregate coarse-to-fine features at the current scale and both the higher scale and the lower scale (**pyramid scales**). Meanwhile, pyramid input enhancement (marked by yellow rectangles) and deep pyramid supervision (marked by purple rectangles) are employed to fuse the original images with corresponding scales, and supervise the intermediate results in each layer of the decoder, respectively.

Intra-layer pyramid-scale aggregation block are based on the ResNet block (43), which is widely adopted in deep learning. Figure 4 illustrates the structure of the ResNet block (43), which is formulated as


(1) $$X_{l+1}=f(X_{l})+X_{l},$$

where *X*_*l*_ and *X*_*l*+1_ are the input and the output of the current layer, while *f*(·) represents the main branch of the current layer. ResNet learns the additive residual function *f*(·) with respect to the unit input through a shortcut connection between them. Meanwhile, the multi-scale feature aggregation inspires us to propose associated branches to learn coarse-to-fine features in each residual branch. Figure 4 illustrates the detailed structures of traditional ResNet blocks and our IPABs. Different from ResNet blocks, in each layer, IPABs generate two associated branches to aggregate coarse-to-fine feature maps to assist the main branch at the current scale. In each branch, the processing steps are almost the same as those in traditional ResNet blocks. Some extra steps such as up-sampling and down-sampling are adopted at the higher and the lower scales to adjust scales. In order to reduce the potential increase of computational cost, the number of channels of the inputs *X*_*l*_ in the main branch has been reduced to half, while the number of channels of resized inputs $X_{l}^{p}$ and $X_{l}^{d}$ in the associated branches is reduced to one-fourth. The feature maps with channel adjustment are fed to the processing steps at three scales and are processed in parallel. The three outputs at pyramid scales are then concatenated. The whole process is formulated as follows,


(2) $${\tilde{X}}_{l+1}=H(f({\hat{X}}_{l}^{p}),f({\hat{X}}_{l}),f({\hat{X}}_{l}^{d}))+X_{l},\;$$

where $X_{l}^{p}$ and $X_{l}^{d}$ are the up-sampled and the down-sampled results of the current input *X*_*l*_ with channel adjustment, respectively. ${\hat{X}}_{l}^{p}$, ${\hat{X}}_{l}$ and ${\hat{X}}_{l}^{d}$ are the enhanced results using pyramid input enhancement, which only exists in the encoder and is detailed in section 3.2. Meanwhile, ${\hat{X}}_{l}^{p}$, ${\hat{X}}_{l}$, and ${\hat{X}}_{l}^{d}$ are replaced by ${\hat{X}}_{l}^{p}$, ${\hat{X}}_{l}$, and ${\hat{X}}_{l}^{d}$ in the decoder, which represents the enhancement results by pyramid skip connections and are detailed in section 3.4. *H*(·) represents the aggregation process, which performs re-scaling and feature concatenation. ${\tilde{X}}_{l+1}$ is the strengthened results of *X*_*l*+1_ by IPAB.

The channel attention module selectively emphasizes interdependent channel maps by integrating associated features among all channel maps. To improve the efficiency of feature extraction, we also employ an attention mechanism (44, 45) in IPAB as follows,


(3) $$\Phi ({\tilde{X}}_{l+1})=Q(\Phi_{Avg}({\tilde{X}}_{l+1}))+Q(\Phi_{Max}({\tilde{X}}_{l+1})).$$

(4) $$\Psi ({\tilde{X}}_{l+1})=\sigma (\Phi ({\tilde{X}}_{l+1})⊗{\tilde{X}}_{l+1}.$$

where Ψ(·) is the operation of attention process, **Q** is the conventional operation using 1×1 kernels for channel adjustment, and σ is the activation function. Average-pooling Φ_*Avg*_(·) and max-pooling Φ_*Max*_(·) are adopted to aggregate channel information. By utilizing IPAB, each layer of the network aggregates the feature with pyramid scales, which helps fuse coarse-to-fine context information to improve the overall segmentation performance.

### 3.2. Pyramid Input Enhancement

Pyramid input enhancement fuses the input image with multiple scales to IPABs to reduce the loss of information caused by re-scaling and enhance feature fusion. Pooling operations with various pooling sizes are used to guarantee spatial resolution consistency. Particularly, in each layer, the input image is scaled at higher, current, and lower scales, and fed to three parallel processing steps at multiple scales in the IPAB. Pooling operations over larger regions successively reinforce the scale and translation invariance while reducing noise sensitivity at the same time as more and more context information is added. The aggregation should facilitate discrimination between relevant features and local noises. The above three pyramid-scale images are concatenated with corresponding outputs of up-sampling, down-sampling, and channel adjustment, respectively. Suppose that *X*_*l*_ is denoted as the input of the current layer, and $X_{l}^{p}$, and $X_{l}^{d}$ are results at the higher scale and the lower scale, respectively. Meanwhile, *I*_*l*−1_, *I*_*l*_ and *I*_*l*+1_ are the scaled inputs of $X_{l}^{d}$, *X*_*l*_, and $X_{l}^{p}$ with the same size, respectively. The fusion process of the current scale is formulated as follows,


(5) $${\hat{X}}_{l-1}=H(X_{l}^{d},W^{d}(I_{l-1})),$$

(6) $${\hat{X}}_{l}=H(X_{l},W(I_{l})),$$

(7) $${\hat{X}}_{l+1}=H(X_{l}^{p},W^{p}(I_{l+1})),$$

where **W**^*p*^(·), **W**^*d*^(·), and **W**(·) represents 3×3 convolutional operations and is applied before concatenating to the pyramid-scale features, and *H*(·) denotes channel adjustment.

### 3.3. Deep Pyramid Supervision

Deep pyramid supervision optimizes feature maps at multiple scales to improve the segmentation of multi-scale objects and fast the training process. Similar to pyramid input enhancement, deep pyramid supervision connects the intermediate layer to the final prediction thus fusing coarse-to-fine context information. Particularly, the feature maps at multiple scales from each IPAB in the decoder are fed into a plain 3 × 3 convolutional layer followed by Sigmoid function. Deep pyramid supervision at the *l*th scale of the decoder can be defined as,


(8) $$L_{l}=L(Y_{l}^{p},M_{l-1})+L(Y_{l},M_{l})+L(Y_{l}^{d},M_{l+1}).$$

The ground truths *M* are scaled to the same size as the pyramid-scale feature maps for deep supervision, e.g., $Y_{l}^{p},Y_{l}$, and $Y_{l}^{d}$ are supervised by the corresponding ground truth *M*_*l*−1_, *M*_*l*_, and *M*_*l*+1_, respectively. Note that the feature maps in each layer can be directly fused with the final prediction and optimized without massive convolutional processing. Therefore, deep pyramid supervision can be adapted to different depths for different tasks in training, which supply adaptive model capacity, thereby facilitating the segmentation of objects with different scales.

### 3.4. Pyramid Skip Connections

Pyramid skip connections perform feature reuse among the three scaled feature maps (the higher scale, the current scale, and the lower scale) in each IPAB module. Suppose that *X*_*l*_ is the input of the current layer in the decoder, and $X_{l}^{p}$, and $X_{l}^{d}$ are the results at the higher scale and the lower scale, respectively. Meanwhile, $\left({\tilde{X}}_{l}^{p},{\tilde{X}}_{l+1},{\tilde{X}}_{l+2}^{d}\right)$, $\left({\tilde{X}}_{l-1}^{p},{\tilde{X}}_{l},{\tilde{X}}_{l+1}^{d}\right)$, and $\left({\tilde{X}}_{l-2}^{p},{\tilde{X}}_{l-1},{\tilde{X}}_{l}^{d}\right)$ are three groups of learned feature maps from the encoder, and feature maps in each group have the same spatial dimension with the corresponding scaled input ${\hat{X}}_{l-1}$, ${\hat{X}}_{l}$, and ${\hat{X}}_{l+1}$, respectively. The fusion process of the current scale is formulated as follows,


(9) $${\hat{X}}_{l-1}=H(X_{l}^{d},H({\tilde{X}}_{l}^{p},{\tilde{X}}_{l+1},{\tilde{X}}_{l+2}^{d})),$$

(10) $${\hat{X}}_{l}=H(X_{l},H({\tilde{X}}_{l-1}^{p},{\tilde{X}}_{l},{\tilde{X}}_{l+1}^{d})),$$

(11) $${\hat{X}}_{l+1}=H(X_{l}^{p},H({\tilde{X}}_{l}^{d},{\tilde{X}}_{l-1},{\tilde{X}}_{l-2}^{p})),$$

where *H*(·) denotes channel adjustment. We can see that features at the current-scale *l* can reuse and aggregate feature maps at most five scales (*l* − 2, *l* − 1, *l, l* + 1, and*l* + 2).

## 4. Experiments

### 4.1. Datasets

We used three public available retinal vessel datasets, DRIVE (20), STARE (21), and CHASE-DB1 (22) for evaluation. The images in the three datasets are collected using digital retinal imaging, a standard method of documenting the appearance of the retina. More details of the datasets are as follows.

**DRIVE:** The DRIVE dataset (20) consists of 40 images with a resolution of 565 × 584 pixels, which were acquired using a Canon CR5 non-mydriatic 3CCD camera with a 45-degree field of view (FOV). Two trained human observers labeled the vessels in all images, and the ones from the first observer were used for network training. The dataset has been divided into a training and a test set (20), both of which contain 20 images.

**CHASE-DB1:** The CHASE-DB1 dataset (22) contains vascular patch images with a resolution of 999 × 960, which were acquired from 28 eyes of 14 ten-year-old children. Since images were captured in subdued lighting and the operators adjusted illumination settings, the images contain more illumination variation in CHASE-DB1 compared with the DRIVE datasets. Following the configuration in Li et al. (46), the first 20 images and the remaining 8 images are employed as the training set and the test set, respectively.

**STARE:** The STARE dataset (21) consists of 20 equal-sized images with a resolution of 700 × 605 pixels. Each image is with a 35° FOV, and half of the images of eyes are with ocular pathology. As the training set and the test set are not explicitly specified, the same leave-one-out cross-validation is adopted (33) for performance evaluation, where models are iteratively trained on 19 images and tested on the rest images. Liking other methods (10), manual annotations generated by the first observer are used for both training and test.

### 4.2. Implementations

All experiments were conducted on an Nvidia GeForce Titan X (pascal) containing 12 GB memory. Meanwhile, we employed CE-Net (32), one of the state-of-the-art methods in retinal vessel segmentation, as the backbone models to implement IPABs, pyramid input enhancement, deep pyramid supervision, and pyramid skip connections. Normalization of the training data has been implemented. In order to express the details of multi-scale feature fusion more clearly, we use U-Net as the basic network to explain, which is widely used in the medical image segmentation domain. In practice, we use the state-of-the-art method CE-Net to replace U-Net to obtain better performance. During training, we adopted Adaptive Moment Estimation (Adam) as the learning optimizer with a batch size of 4. Data augmentation operations including horizontal flip, vertical flip, and diagonal flip are used to enlarge the train samples. We use a threshold to obtain the final segmentation from pixel probability vectors. Particularly, the pixels with values smaller than the threshold are assigned to the background class, and the remaining pixels with values equal to or greater than the threshold are categorized as the vessel class. The final prediction is the ensemble of the segmentation output of the vessel images, its rotation (90°), and its flip (horizontal and vertical).

### 4.3. Evaluation Metrics

We introduce four evaluation metrics including Sensitivity (Sens), Specificity (Spec), Accuracy (Acc), and Area Under the ROC Curve (AUC) to validate our proposed Pyramid-Net. The metrics are calculated as follows:


(12) Sensitivity=TP/(TP+FN),

(13) Specificity=TN/(TN+FP),

(14) Accuracy=(TP+TN)/(TP+TN+FP+FN).

True positive (TP) and true negative (TN) present that pixels are correctly classified to objects or backgrounds, respectively. Meanwhile, pixels will be labeled as false positive (FP) or false negative (FN), if they are misclassified to objects or backgrounds, respectively.

### 4.4. Quantitative Results

We compared our Pyramid-Net with existing state-of-the-art works on three vessel image segmentation datasets (DRIVE, CHASE-DB1, and STARE). Tables 1–3 illustrate the comparison results of Pyramid-Net and the current state-of-the-art methods. For the DRIVE dataset, Pyramid-Net achieves a high score of 82.38, 98.19, 96.26, and 98.32% on Sens, Spec, Acc, and AUC, respectively, and outperforms state-of-the-art methods in three metrics including Spec, Acc, and AUC. In terms of Sens, CE-Net achieves the best performance of 83.09%, while our method achieves a comparable result, which is 0.71% lower. Overall, Pyramid-Net achieves higher overall performance than CE-Net. For the CHASE-DB1 dataset, compared with the state-of-the-art results, the proposed Pyramid-Net achieves high score of 81.17, 98.26, 96.89, and 98.92% for Sens, Spec, Acc, and AUC, respectively, which consistently enjoys a better performance than all the current state-of-the-art methods. For the STARE dataset, Pyramid-Net achieves a promising score of 82.35, 98.87, 97.19, and 98.62% for Sens, Spec, Acc, and AUC, respectively, which is also consistently better than all the current state-of-the-art methods. The consistent improvements in Tables 1–3 indicate the effectiveness and robustness of our Pyramid-Net.

**Table 1:** Performance comparison of Pyramid-Net and the state-of-the-art methods on the DRIVE dataset.

**Method**	**Sens (%)**	**Spec (%)**	**Acc (%)**	**AUC (%)**
FCN (31)	74.89	96.21	94.13	95.67
U-Net (23)	75.31	96.45	94.45	96.01
DeepVessel (11)	76.12	97.68	95.23	97.52
(10)	76.53	98.18	95.42	97.52
(47)	77.92	98.13	95.56	97.84
(40)	78.44	98.07	95.67	98.19
CE-Net (32)	83.09	97.47	95.45	97.79
BTS-DSN (48)	78.91	98.04	95.61	98.06
(49)	79.16	98.11	95.70	98.10
(50)	79.40	98.16	95.67	97.72
Vessel-Net (51)	80.38	98.02	95.78	98.21
MResU-Net (52)	79.69	97.99	-	97.99
CTF-Net (53)	78.49	98.13	95.67	97.88
Hybrid-Net (6)	**83.53**	97.51	95.79	-
HA-Net (33)	79.91	98.13	95.81	98.23
**Pyramid-Net**	82.38	**98.19**	**96.26**	**98.32**

*Bold values mean the state-of-the-art performance*.

**Table 2:** Performance comparison of Pyramid-Net and the state-of-the-art methods on the CHASE-DB1 dataset.

**Method**	**Sens (%)**	**Spec (%)**	**Acc (%)**	**AUC (%)**
(54)	76.15	95.75	94.67	96.23
(46)	75.07	97.93	95.81	97.16
(55)	81.94	97.39	96.30	-
(10)	76.33	98.09	96.10	97.81
(47)	77.56	98.20	96.34	98.15
FCN (31)	76.41	98.06	96.07	97.76
(56)	81.55	97.52	96.10	98.04
(48)	78.88	98.01	96.27	98.40
(50)	80.74	98.21	96.61	98.12
(51)	81.32	98.14	96.61	98.60
Three-stage (12)	76.41	98.06	96.07	97.76
CTF-Net (52)	79.48	**98.42**	96.48	98.47
Hybrid-Net (6)	81.76	97.76	96.32	-
HA-Net (33)	**82.39**	98.13	96.70	98.70
Pyramid-Net	81.17	98.26	**96.89**	**98.92**

*Bold values mean the state-of-the-art performance*.

**Table 3:** Performance comparison of Pyramid-Net and the state-of-the-art methods on the STARE dataset.

**Method**	**Sens (%)**	**Spec (%)**	**Acc (%)**	**AUC (%)**
(54)	73.20	98.40	95.60	96.70
(57)	77.91	97.58	95.54	97.48
(58)	76.80	97.38	-	-
(10)	75.81	98.46	96.12	98.01
(56)	75.95	98.78	96.41	98.32
Three-stage (12)	77.35	98.57	96.38	98.33
MResU-Net (52)	81.01	97.95	-	98.16
Hybrid-Net (6)	79.46	98.21	96.26	-
HA-Net (33)	81.86	98.44	96.73	98.32
**Pyramid-Net**	**82.35**	**98.87**	**97.19**	**98.62**

*Bold values mean the state-of-the-art performance*.

### 4.5. Qualitative Results

**Figure 5:**
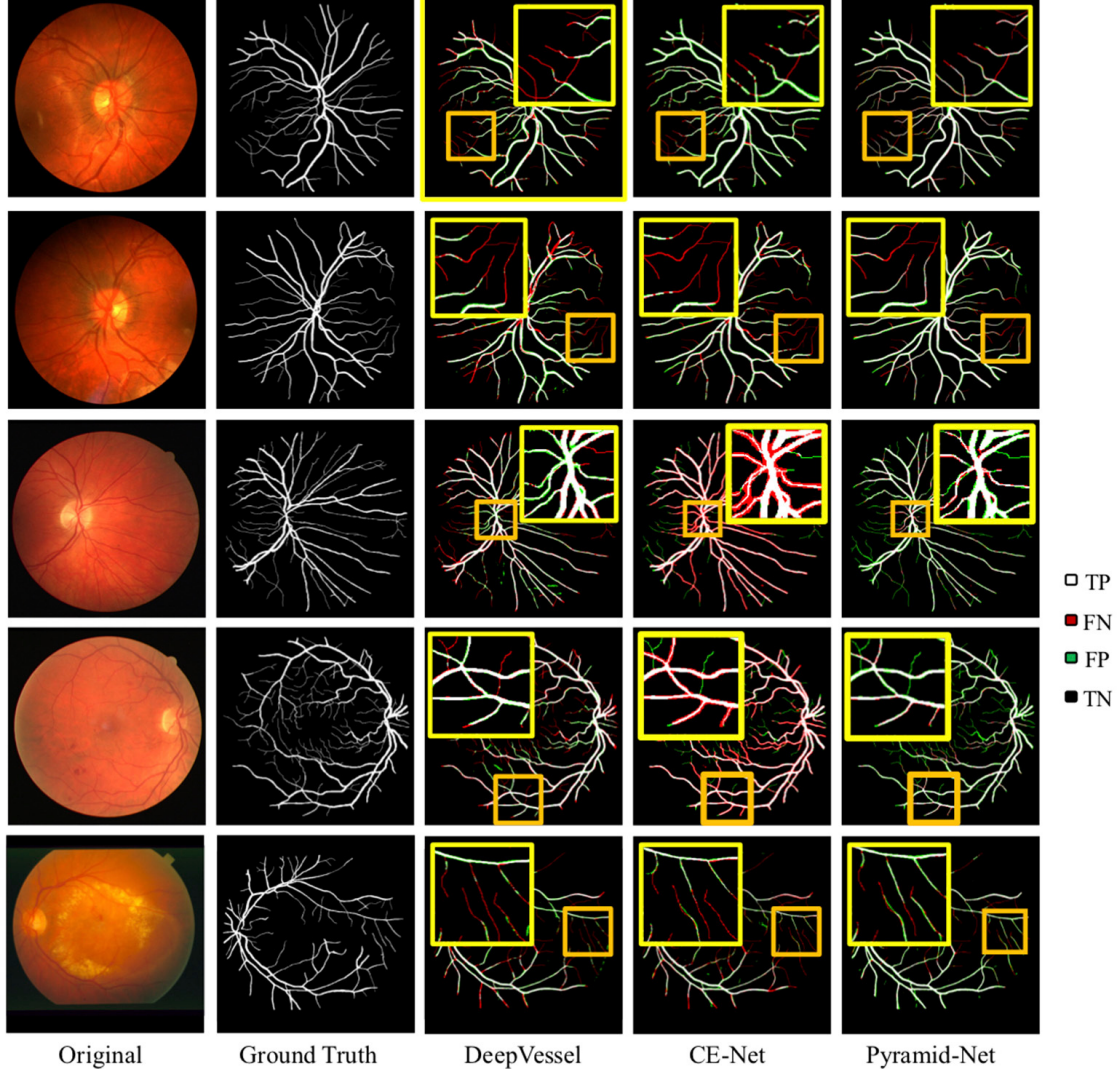
Visual comparison of Pyramid-Net and the state-of-the-art methods including DeepVessel (11) and CE-Net (32) on DRIVE (Row 1–2), CHASE-DB1 (Row 3–4), and STARE (Row 5) datasets. White (TP) and black (TN) pixels indicate correct predictions of object and background, respectively, while red (FP) and green (FN) pixels indicate incorrect predictions. The dark yellow rectangle contains the area used to compare segmentation details, and the bright yellow rectangle contains the zoomed area in the dark yellow rectangle. Best viewed in color.

The visual comparisons between Pyramid-Net and the state-of-the-art methods, including DeepVessel and CE-Net on the DRIVE dataset and the CHASE-DB1 dataset are shown in Figure 5. White (TP) and black (TN) pixels are correct predictions of vessels and the background, respectively, while red (FP) and green (FN) pixels are incorrect predictions. In Figure 5, dark yellow rectangles contain the selected areas used for detail comparison, and the bright yellow rectangles contain the zoomed area in the dark yellow rectangle. We can notice that current methods enjoy a good performance on the segmentation of main retinal vessels, but the effect on some capillaries is poor. For example, Row 1 of Figure 5 shows that the result of DeepVessel misses a large number of thin vessels on the DRIVE dataset, and that of CE-Net obtains a much better accuracy on thin vessels. However, in Row 2, there is no significant difference between the results of the two methods. In both Rows 1 and 2 of Figure 5, our method can achieve much higher accuracy, but we can still notice that our method cannot segment them correctly if the vessels are too thin. We can further observe that our method has much fewer false-negative pixels (indicated by green) than the other two. This may due to the fact that our proposed IPABs can consider more scales thus improving the segmentation accuracy. Overall, our proposed Pyramid-Net evidently improves the segmentation performance, especially for those narrow, low-contrast, and ambiguous retinal vessels.

### 4.6. Evaluation on Thin Vessels

In the previous subsection, the results in Figure 5 indicate that though the main vessels enjoy a promising segmentation performance, the segmentation of thin vessels always suffers a big miss in the prediction. In practice, it is challenging to segment the thin vessels from the complex retina background, which are always low-contrast and extremely narrow (1–2 pixels). Thus, in this subsection, to evaluate the effectiveness of Pyramid-Net on thin vessels, we compared Pyramid-Net with the state-of-the-art methods on an additional dataset only containing thin vessel labels. Vessels with a width of 1 or 2 pixels are commonly regarded as the thin vessels in the DRIVE dataset. To avoid potential unfair in the evaluation on the manual addition label of the thin vessel, we distinguish thick vessels from thin vessels by an opening operation (10). The evaluation results are summarized in Table 4. It can be noticed that Pyramid-Net achieves a high ACC score of 96.26, 96.51, and 91.64% on all vessels, thick vessels, and thin vessels, respectively. Overall, our method outperforms the state-of-the-art methods on all metrics. As for the thin vessel segmentation, our methods achieve an improvement of 4.73% over backbone model CE-Net and outperforms the state-of-the-art method by about 3.86%. The experiment results indicate that our Pyramid-Net is particularly effective on thin vessels.

**Table 4:** Performance comparison on thick and thin vessels of Pyramid-Net on the DRIVE dataset.

**Method**	**All vessel (%)**	**Thick vessel (%)**	**Thin vessel (%)**
(10)	95.42	95.78	87.78
CE-Net (32)	95.45	95.96	86.91
**Pyramid-Net**	**96.26**	**96.51**	**91.64**

*Bold values mean the state-of-the-art performance*.

### 4.7. Ablation Analysis

To justify the effectiveness of IPABs, pyramid input enhancement, deep pyramid supervision, and pyramid skip connections in the proposed Pyramid-Net, we conduct ablation analysis using the DRIVE dataset as a vehicle. The ablation experimental results are summarized in Table 5. We use CE-Net (32) as our backbone, which achieves a good score of 95.45 and 97.79% on Acc and on AUC, respectively. Firstly, we evaluate the effectiveness of IPABs on the backbone. Benefiting from aggregating coarse-to-fine context information from pyramid scale in each layer, the backbone model with IPABs achieves improvements of 0.62% on Acc and 0.30% on AUC. Second, we evaluate pyramid input enhancement and deep pyramid supervision to feed the original image at multiple scales into the network and supervise the immediate layers contains features at various scales. In Table 5, we can notice that the above two optimizations achieve improvements of more than 0.10 and 0.07% in AUC, respectively. Third, pyramid skip connections connect the encoder and the decoder and make full use of the features from multiple layers and scales in the encoder, which achieves an improvement of about 0.15% on AUC. Overall, integrating the pyramid-scale concept into the design of the basic unit and skip connections can obviously improve the network segmentation, and the other two optimizations also bring some improvement.

**Table 5:** Ablation analysis of Pyramid-Net on the DRIVE dataset.

**Method**	**Acc (%)**	**AUC (%)**
Baseline	95.45	97.79
Baseline + IPABs	96.07	98.09
Baseline + IPABs + pyramid input	96.10	98.15
Baseline + IPABs + Pyramid supervision	96.15	98.12
Baseline + IPABs + pyramid skip connection	96.21	98.24
**Pyramid-Net**	**96.26**	**98.32**

*Bold values mean the state-of-the-art performance*.

### 4.8. Cross-Training Evaluation

To evaluate the generalization of Pyramid-Net, we performed a cross-training evaluation on the DRIVE dataset and the STARE dataset. We directly implemented our models trained on the source dataset and tested on the target dataset for fair comparisons. The experimental results are summarized in Table 6. Overall, our method achieves the state-of-the-art transfer performance on both configurations. Particularly, for the configuration that models are trained on the STARE dataset and tested on the DRIVE dataset, it can be noticed that the transfer model can achieve competitive results on Spec and suffer a big loss of accuracy on Sens. The potential reason is the imbalance between thick vessels and thin vessels in the STARE dataset. Manual annotations of the STARE dataset contain more thick vessels than thin vessels, which led that the pre-trained model on the STARE dataset obtains a bad segmentation performance of thin vessels on the DRIVE dataset. When the conditions are reversed, the above situation is alleviated, and the corresponding scores on Sens, Spec, Acc, and AUC on the STARE dataset are comparable with the model trained on the STARE dataset.

**Table 6:** Cross-training evaluation on the DRIVE dataset and the STARE dataset.

**Method**	**Sens (%)**	**Spec (%)**	**Acc (%)**	**AUC (%)**
**DRIVE (train) ->** **STARE (test)**				
(12)	70.14	98.02	94.44	95.68
(56)	65.05	99.14	94.81	97.18
HA-Net (33)	71.40	98.79	95.30	97.58
Pyramid-Net	**75.71**	**98.86**	**95.57**	**97.78**
**STARE (train) -> DRIVE (test)**				
(12)	73.19	98.40	95.80	96.78
(56)	70.00	97.59	94.74	97.18
HA-Net (33)	81.87	**98.79**	95.30	97.58
Pyramid-Net	**82.67**	98.76	**95.36**	**97.72**

*Bold values mean the state-of-the-art performance*.

### 4.9. Comparison With Multi-Scale Aggregation Methods

To evaluate the effectiveness of the multi-scale information aggregated in the proposed Pyramid-Net, we compare existing multi-scale aggregation methods, including Dense Pooling Connections (15), Complete Bipartite Network (CB-Net) (16), Dense Decoder Short Connections (DDSC) (18), and U-Net++ (17) on the DRIVE dataset. For fair comparisons, we directly implement those different connection styles and our Pyramid-Net on U-Net (23). The comparison results and the *p*-values for the paired *t*-test are summarized in Table 7. Compared with existing methods, our method outperforms them by 0.65–0.99% and 0.67–1.50% on Acc and AUC, respectively. On the other hand, we also compare the computational cost of the proposed Pyramid-Net with existing methods. Obviously, existing methods improve the network performance and increase the computational cost by 16.38–493.74G (104.9–247.4%) on FLOPs from the numerous feature reuse. Particularly, our proposed Pyramid-Net achieves state-of-the-art performance with a computational cost reduced by 216.8G (64.7%) on FLOPs. The reason for the above phenomenon is the channel reduction in each IPAB. The channels' main branch is reduced to half, while the number of channels at associated branches is half of that of the main branch. Overall, our method achieves the state-of-the-art performance of 96.26% on Acc and 98.32% on AUC with a 64.7% reduction on FLOPs.

**Table 7:** Comparison with existing multi-scale aggregation methods on the DRIVE Dataset.

**Method**	**Acc (%)**	**AUC (%)**	**FLOPs**	***p*-values**
U-Net (23)	94.45	96.01	334.95G	<0.01
DPC (15)	95.56	97.65	351.33G	<0.01
CB-Net (16)	95.61	97.52	441.62G	<0.01
DDSC (18)	95.42	97.48	381.07G	<0.01
U-Net ++ (17)	95.27	96.82	828.69G	<0.01
CE-Net (32)	95.45	97.79	-	<0.05
**Pyramid-Net**	**96.26**	**98.32**	188.15G	-

*Bold values mean the state-of-the-art performance*.

## 5. Conclusion

In this paper, we introduced Pyramid-Net for accurate retinal vessel segmentation. In Pyramid-Net, the proposed IPABs are utilized to generalize two associated branches to aggregate coarse-to-fine feature maps at pyramid scales to improve the segmentation performance. Meanwhile, three optimizations including pyramid inputs enhancement, deep pyramid supervision, and pyramid skip connections are implemented with IPABs in the encoder, the decoder, and the cross of the two to further improve performance, respectively. Comprehensive experiments have been conducted on three retinal vessel segmentation datasets, including DRIVE (20), STARE (21), and CHASE-DB1 (22). Experimental results demonstrate that our IPABs can efficiently improve the segmentation performance, especially for thin vessels. In addition, our method is also much more efficient than existing methods with a large reduction in computational cost.

## Data Availability Statement

The original contributions presented in the study are included in the article/supplementary material, further inquiries can be directed to the corresponding authors/s.

## Author Contributions

XX is the guarantor of the manuscript. JZh implemented the experiments and wrote the first draft of the manuscript. HQ, WX, and ZY managed the result analysis. All authors contributed to drawing up the manuscript.

## Funding

This work was supported by the National Key Research and Development Program of China (no. 2018YFC1002600), the Science and Technology Planning Project of Guangdong Province, China (nos. 2017B090904034, 2017B030314109, 2018B090944002, and 2019B020230003), Guangdong Peak Project (no. DFJH201802), and the National Natural Science Foundation of China (no. 62006050).

## Conflict of Interest

The authors declare that the research was conducted in the absence of any commercial or financial relationships that could be construed as a potential conflict of interest.

## Publisher's Note

All claims expressed in this article are solely those of the authors and do not necessarily represent those of their affiliated organizations, or those of the publisher, the editors and the reviewers. Any product that may be evaluated in this article, or claim that may be made by its manufacturer, is not guaranteed or endorsed by the publisher.
